# Cancer Stem Cell Plasticity Drives Therapeutic Resistance

**DOI:** 10.3390/cancers8010008

**Published:** 2016-01-05

**Authors:** Mary R. Doherty, Jacob M. Smigiel, Damian J. Junk, Mark W. Jackson

**Affiliations:** 1 Department of Pathology, School of Medicine, Case Western Reserve University, 2103 Cornell Road, Cleveland, OH 44106, USA; mrd85@case.edu (M.R.D.); jxs1094@case.edu (J.M.S.); damian.junk@case.edu (D.J.J.); 2 Case Comprehensive Cancer Center, Case Western Reserve University, 10900 Euclid Ave, Cleveland, OH 44106, USA

**Keywords:** cancer stem cells, therapeutic resistance, cellular plasticity, epithelial-mesenchymal, tumor microenvironment, cytokines

## Abstract

The connection between epithelial-mesenchymal (E-M) plasticity and cancer stem cell (CSC) properties has been paradigm-shifting, linking tumor cell invasion and metastasis with therapeutic recurrence. However, despite their importance, the molecular pathways involved in generating invasive, metastatic, and therapy-resistant CSCs remain poorly understood. The enrichment of cells with a mesenchymal/CSC phenotype following therapy has been interpreted in two different ways. The original interpretation posited that therapy kills non-CSCs while sparing pre-existing CSCs. However, evidence is emerging that suggests non-CSCs can be induced into a transient, drug-tolerant, CSC-like state by chemotherapy. The ability to transition between distinct cell states may be as critical for the survival of tumor cells following therapy as it is for metastatic progression. Therefore, inhibition of the pathways that promote E-M and CSC plasticity may suppress tumor recurrence following chemotherapy. Here, we review the emerging appreciation for how plasticity confers therapeutic resistance and tumor recurrence.

## 1. Introduction

The metastatic spread of cancer cells and tumor recurrence following therapy causes 90% of cancer mortalities [1,2,3,4,5]. However, despite their importance, our understanding of the molecular pathways involved in generating invasive, metastatic and therapy-resistant cells remains fairly limited. Tumors are complex tissues composed of a hierarchical organization of tumor cell sub-populations as well as numerous tumor-associated stromal cell types [6]. The cancer stem cell (CSC) hypothesis places cells with self-renewal capacity and the ability to differentiate at the top of the tumor cell hierarchy [7,8,9,10]. By definition, CSCs have the ability to form tumors that recapitulate the heterogeneity of the primary tumor from which they were isolated following orthotopic transplantation into mice [11,12]. As the progeny of CSCs differentiate, they lose their ability to generate tumors, despite their identical genetic landscape. This highlights the importance of the tumor microenvironment (TME), which serves as the CSC niche, to regulate CSC self-renewal and differentiation [13,14]. Current evidence supports the hypothesis that CSCs harbor properties that promote therapeutic resistance (upregulation of drug efflux pumps, activation of signaling that circumvents drug targets, and deregulation of apoptotic signaling). In essence, CSCs are the “roots” of aggressive tumors for which we currently have no effective treatments. Devising a way to kill CSCs or prevent them from being generated is a significant clinical challenge. Recent evidence suggests that not only do CSCs exist in tumors prior to treatment, but that treatment can also induce the *de novo* generation of cells harboring CSC properties. The degree to which the cells that acquire CSC properties in response to chemotherapy are similar to those found in untreated tumors is an important question, yet it remains undetermined. Thus, we will refer to non-CSC cells that have acquired CSC properties as “CSC-like”. Here, we review how cells harboring CSC properties contribute to therapeutic resistance and discuss the emerging evidence suggesting that non-CSC cells can acquire these key, CSC properties.

## 2. Connecting the Dots: CSC Properties and Epithelial-Mesenchymal Transition (EMT)

CSCs have been isolated from nearly every type of human malignancy using a limited (albeit non-overlapping) set of markers [7]. For example, breast CSCs were originally identified as having a CD24^Lo^CD44^Hi^ cell-surface marker profile [11]. Subsequently, elevated Aldehyde Dehydrogenase (ALDH) activity was also shown to correlate with CSC potential, although CD24^Lo^CD44^Hi^ and ALDH+ CSCs identify distinct CSC populations [15,16]. Thus, rather than using specific markers, functional assays are necessary to truly define a CSC population, including the capacity for anchorage-independent growth (AIG) as tumorspheres and the ability to generate tumors following orthotopic implantation (typically at low cell numbers; [12]). Our studies, along with the studies of others, have determined that transformed human mammary epithelial cells (HMEC) acquire CSC properties when they undergo EMT, in addition to acquiring an invasive, mesenchymal phenotype [9,17,18,19,20,21].

The acquisition of mesenchymal/CSC properties can occur spontaneously during the transformation process, following exogenous expression of EMT-inducing transcription factors, or upon exposure to specific cytokines. For example, the induction of EMT in immortalized and transformed human mammary epithelial cells (HMEC) by ectopic expression of Twist, Snail, or FoxC2 transcription factors induces EMT, concomitant with an increased ability to form tumorspheres *in vitro* and tumors *in vivo* [19,22,23]. Our laboratory has demonstrated that transformed HMEC harboring a mesenchymal phenotype arose spontaneously during the transformation process [24]. However, the spontaneously generated mesenchymal cells lack plasticity. In contrast, exposure of non-CSCs to Transforming Growth Factor Beta (TGF-β) generated a mesenchymal/CSC-like population, which retained remarkable plasticity. The TGF-β-induced CSC-like cells required the sustained presence of TGF-β, as removal of TGF-β or inhibition of TGF-β signaling (by pharmacologic or genetic means) led to a marked loss in mesenchymal/CSC properties and the re-emergence of an epithelial/non-CSC phenotype (or differentiation). We propose that targeting cytokine signaling emanating from the TME (such as TGF-β) may disrupt E-M plasticity and inhibit the emergence of invasive, drug-resistant CSC-like cells. The link between E-M plasticity and CSC properties has been paradigm-shifting, coupling key concepts that relate metastatic progression with therapeutic resistance resulting in tumor recurrence. We posit that the TME, acting as the CSC niche, serves as the key source of plasticity-inducing cytokines. In fact, beyond TGF-β, many TME cytokines are emerging that can induce EMT and CSC properties. Further characterization of the cytokines present in the TME and defining how they impact E-M and CSC plasticity is of key importance for understanding metastasis and recurrence.

## 3. Mirroring Metastasis: Trends in Tumor Recurrence

Metastasis is the overwhelming cause of cancer mortality [1,2]. Despite recent studies linking CSC properties with metastatic progression, our understanding of this complex problem remains limited. Metastasis is most often thought to be initiated by epithelial cancer cells undergoing EMT, resulting in the dissolution of tight cell-cell interaction by downregulation of junctional/epithelial proteins (claudin, occludin, ZO1, E-cadherin, cytokeratins; [25,26]). EMT is regulated by transcription factors that include Snail, Twist, and ZEB1/2, which initiate the repression of the epithelial phenotype and activation of mesenchymal markers (N-cadherin, Vimentin). The loss of important cell-cell interactions is an important step in allowing escape from the primary tumor and entrance into the lymphatics or bloodstream, but additional characteristics that facilitate the metastatic cascade are also acquired during EMT. These include: (1) the induction of matrix metalloproteinases (MMPs) responsible for the degradation of the extracellular matrix (ECM) and tumor cell intravasation; (2) survival in the bloodstream (by creating multi-cell aggregates); and (3) extravasation (by enhanced membrane protrusions and blood vessel contacts [27]).

EMT in primary Triple Negative Breast Cancer (TNBC), as evidenced by loss of epithelial markers (E-cadherin) or gain of mesenchymal markers (Vimentin, N-Cadherin, Snail expression) typically at the tumor’s edge, correlates with poor clinical outcome in TNBC [28,29,30,31,32]. Likewise, the presence and abundance of circulating tumor cells (CTC) that express mesenchymal markers can be used to track a patient’s response to therapy [33,34,35]. For example, patients that respond positively to therapy have a decrease in mesenchymal CTC while patients with progressive disease have an increase in mesenchymal CTC following treatment [34]. Moreover, the re-emergence of mesenchymal CTC in initial responders is accompanied by tumor recurrence. Further evidence linking E-M plasticity and a CSC phenotype is provided by the observation that a sub-set of CTC responsible for initiating metastasis (termed the metastasis-initiating cells) express high levels of the CSC marker CD44 or ALDH1 [36,37,38]. Moreover, single-cell analysis recently identified distinct EMT and CSC gene expression patterns in early-stage breast cancer micro-metastases [39]. Importantly, larger, late-state metastases were more heterogeneous, more proliferative, expressed higher levels of differentiation markers, and displayed similarity to the primary tumors. Since these micro- and macro-metastases were identified from the same patient-derived xenografts, the findings are consistent with the idea that mesenchymal/CSCs initiate metastatic outgrowth at a secondary site, followed later by increased proliferation and differentiation. Likewise, evidence has suggested that epithelial cancer cells, which undergo EMT to initiate metastases, must undergo a reciprocal mesenchymal-epithelial transition (MET) at secondary sites, as macro-metastases have long been noted to have a largely epithelial phenotype [27]. The conditions regulating E-M and CSC plasticity at secondary sites are currently unclear, but may be due to the significant change in microenvironmental factors, including the availability of microenvironmental cytokines.

**Figure 1:**
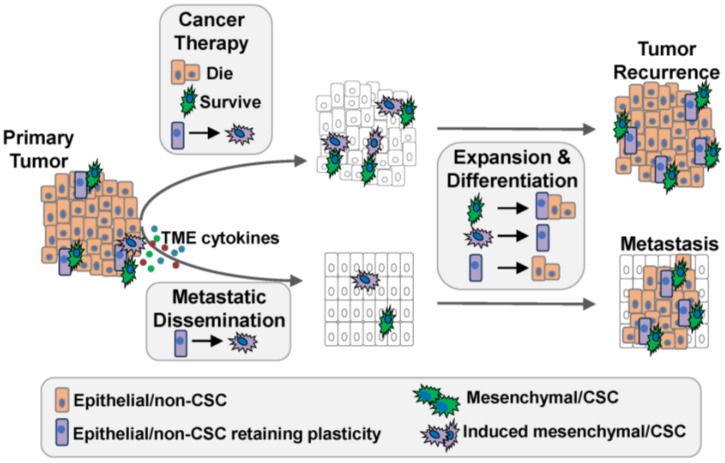
The importance of E-M and CSC plasticity in tumor recurrence and metastatic progression. Following cancer therapy, many non-CSCs are eliminated while select non-CSCs acquire mesenchymal/CSC properties. Together with surviving CSCs, the induced mesenchymal/CSCs grow to establish a recurrent tumor (upper). Similarly, upon metastatic dissemination, CSCs acquire proliferative capacity and differentiate to establish a metastatic tumor (lower).

The idea that tumor cells capable of seeding metastasis must retain (or regain) the ability to proliferate and differentiate mirrors what happens during tumor recurrence following chemotherapy (Figure 1). Differentiated, non-CSCs, which make up the bulk of a tumor, are more sensitive to conventional chemotherapies than CSCs are [7,20,40]. The efficient killing of large numbers of non-CSCs explains the initial responses often observed following treatment with first-line therapy. In contrast, CSCs are more refractory to genotoxic therapies, allowing them to survive, proliferate, and differentiate in order to fuel tumor recurrence. Indeed, analysis of paired breast cancer core biopsies before and after chemotherapy demonstrates that a higher percentage of CD24^Lo^CD44^Hi^ CSCs capable of forming mammospheres exist in post-treatment biopsies [41]. In addition, gene expression analyses of paired breast cancer biopsies before and after endocrine therapy or chemotherapy demonstrate that residual, surviving cells harbor elevated CSC gene expression signatures. CSCs avoid therapy-induced apoptotic signaling through more effective DNA damage responses evolved to protect genomic integrity [42,43]. CSCs also express lower levels of pro-apoptotic proteins, such as caspase-8, and elevated levels of drug efflux pumps, such as MDR-1 and the p-glycoproteins [44], which transport chemotherapeutics out of the cell, rendering them ineffective [45,46].

## 4. *De Novo* Generation of CSC from non-CSC

The enrichment of cells with a CSC phenotype following therapy has been interpreted in two significantly different ways. The original interpretation posited that therapy kills non-CSCs while sparing pre-existing CSCs. However, accumulating evidence supports the idea that non-CSCs can be induced into a drug-tolerant, CSC-like state by chemotherapy (Figure 1; [21,47,48,49,50,51,52]). For example, drug-sensitive Non-Small Cell Lung Carcinoma Cells (NSCLC), which lacked CSC properties prior to therapy, acquired CSC properties following acute exposure to either conventional chemotherapy (paclitaxel) or targeted therapies (Erlotinib). Importantly, this drug-induced CSC-like phenotype was transient, as drug removal resulted in loss of CSC properties as cells differentiated into non-CSCs and regained drug sensitivity [51]. Overall, this observation suggests that CSC plasticity, in response to cancer therapy, can be a transient event engaged during the stress of drug exposure. Interestingly, drug-induced CSC plasticity was dependent on increased expression of epigenetic modifiers such as histone deacetylases (HDAC) as well as growth factor signaling through the Insulin Growth Factor Receptor (IGFR), as pharmacological inhibition of either HDAC or IGFR disrupted CSC plasticity and restored drug sensitivity. The effectiveness of IGFR and HDAC inhibitors was dependent on the timing of their administration, and was only effective in suppressing CSC plasticity when given as an adjuvant therapy. Simply pre-treating with these pharmacological inhibitors prior to conventional therapy was ineffective, demonstrating that the pathways which induced CSC properties were not engaged prior to therapy and thus were not a function of a pre-existing CSC state.

Similarly, emerging evidence in breast cancer suggests that conventional cancer therapy can directly induce *de novo* generation of transient CSC-like cells from non-CSCs and that this CSC plasticity drives acquired therapeutic resistance [47]. In these studies, high-dose exposure of tumor explants and cancer cell lines to taxanes or anthracyclines not only induced cell death in the majority of cells but also induced a phenotypic cell state transition in which some non-CSCs acquired CSC properties. These CSC properties included induction of breast CSC cell surface marker expression, enhanced tumor growth and decreased survival of patient-derived xenograft (PDX) mouse models. Importantly the drug-resistant, CSC-like state was not simply the result of a selective enrichment of pre-existing CSCs, as acute low dose exposure to chemotherapy resulted in a dose-dependent increase in breast CSC-like cells in the absence of cell death. Interestingly, this finding was not exclusive to breast cancer alone, as melanoma, ovarian, and prostate cancer cell lines also responded to conventional cancer therapy with the induction of a chemo-refractory CSC-like population. In the case of breast cancer, however, the *de novo* generation of chemo-refractory CSC-like cells was dependent on SRC kinase signaling, as treatment with the SRC kinase inhibitor Dasatinib re-sensitized the resistant cells to chemotherapy, resulting in decreased cell viability and decreased tumor volume. Importantly, the ability to re-sensitize these resistant cells was again temporally dependent, and only effective following chemotherapy, not as a pre- or co-treatment [47]. Finally, carboplatin and ionizing radiation can also induce the emergence of CSC properties from non-CSCs, which is dependent on stem cell markers Sox2 and Oct3/4 [53,54]. The ability of cancer cells to transition between distinct cell states has prompted deeper thought beyond the paradigm that treatment induces the selection for a pre-existing sub-population. If the induction of CSC properties by cancer therapy is critical for the survival of tumor cells, then targeting the plasticity needed to acquire CSC properties will be an important therapeutic target rather than simply only targeting the CSC state.

## 5. The TME; Supporting E-M and CSC Plasticity

Progeny emanating from CSCs depend not only on the genetic profile of the cell, but also, perhaps more importantly, on the TME surrounding the cell, which serves as the CSC niche [13,14,55]. As a tumor develops, changes occur not only in the tumor cells, but also in nearby tumor-associated stromal cells. Studies of breast tumor interstitial fluid have identified over 1000 distinct proteins present only in the TME [56]. Many of these proteins are secreted from the 20 or more different cell types that are present within the TME [6]. Cytokines top the list of tumor-associated secreted factors, and are likely to have important effects on the pathways that govern CSC and E-M plasticity. In addition to TGFβ, additional cytokines and growth factors (IL4, IL6, OSM, IL10, TNFα, ILEI, IFNγ, and VEGF) have also been implicated in inducing EMT (and by extension, CSC properties; [57,58,59,60,61,62]). The ability of these cytokines and growth factors to induce CSC properties concomitant with EMT may explain why their presence in the TME correlates with poor patient outcomes.

For example, our laboratory has studied how Oncostatin M (OSM) induces aggressive cancer cell properties implicated in poor patient outcomes. Elevated levels of OSM in the TME are associated with highly aggressive metastatic cancers and an increased risk of tumor recurrence (chemotherapy resistance) in a variety of tumor types [61,63,64,65,66,67,68,69,70,71,72,73,74,75,76,77,78,79,80,81,82,83]. OSM in the TME may originate from immune cells, adipose tissue, as well as cancer cells [84,85,86,87,88,89]. Immune cells (especially macrophages) localized at the advancing, infiltrative margins of carcinomas secrete elevated levels of OSM [84]. Moreover, DNA damaging chemotherapy induces additional OSM secretion from macrophages, potentially exacerbating the aggressive properties associated with mesenchymal and CSC properties following treatment with genotoxic therapies [83,90,91]. At the same time, cancer cells from more aggressive tumor types express markedly higher levels of OSM Receptor (OSMR) [64,77,78,92]. Expression of OSMR correlates with the increased expression of the CSC marker CD44 and mesenchymal markers, most frequently at the invasive edge of the tumor near the OSM-secreting macrophages [84,89]. Moreover, exposure of cancer cells or pre-malignant HMEC to OSM is sufficient to induce EMT, acquisition of CSC properties, and an invasive and transformed phenotype [61,63]. OSM serves as but one example of how TME cytokines might contribute to tumor cell survival following therapy, and ultimately, therapeutic resistance and tumor recurrence.

**Figure 2:**
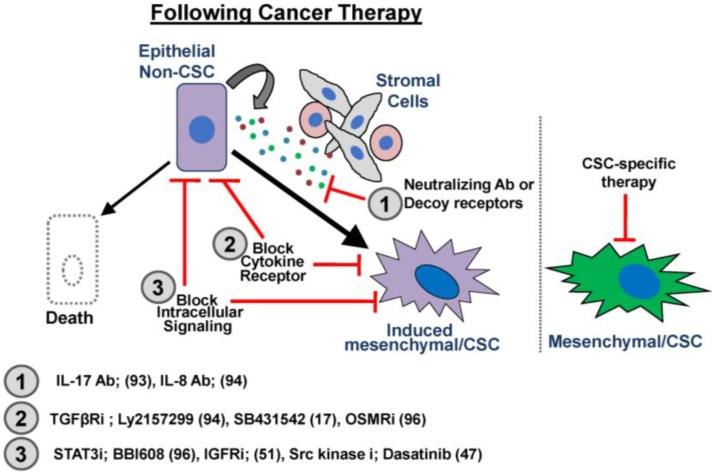
Potential therapeutic targets of CSC plasticity. Cancer therapy induces cell death in many non-CSCs. In select non-CSCs retaining the ability to acquire mesenchymal/CSC properties, autocrine or paracrine (from tumor-associated stromal cells) TME cytokines induce EMT and acquisition of a CSC phenotype. Potential therapeutic strategies to suppress the *de novo* generation of CSCs and prevent therapeutic resistance include (1) the use of neutralizing antibodies (IL-17 Ab); (93), (IL-8 Ab); (94) or decoy receptors to prevent signal initiation in the non-CSCs; (2) blockade of receptor function on the non-CSCs or CSCs (TGFβR inhibitor; LY2157299); (94), SB431542; (17), (OSMR inhibitor); (96); (3) blockade of intracellular signaling emanating from TME cytokine and growth factor receptor activation on the non-CSCs or CSCs (STAT3 inhibitor; BBI608); (96), (IGFR-1 inhibitor); (51), (Src kinase inhibitor; Dasatinib); (47). Targeting the induced CSCs that emerge following cancer therapy would provide a unique approach to limiting tumor recurrence compared to targeting stable CSCs.

Recent comparison of matched patient-derived colorectal cancer samples before and after chemotherapy identified a significant increase in the secretion of IL-17A cytokine from Cancer Associated Fibroblasts (CAFs), which induced CSC properties, resulting in therapeutic resistance [93]. Importantly, pharmacological suppression of IL-17A signaling impaired self-renewal and tumor growth and thus demonstrated that aberrant TME signaling induced by conventional cancer therapy plays an important role in influencing the acquisition of CSC. In addition, paclitaxel treatment was recently shown to induce the expression of a TGFβ gene signature and CSCs in primary patient-derived Triple Negative Breast Cancer (TNBC) tumor biopsies. The expression of the TGFβ signature correlated with increased tumor relapse in treated patients. Importantly, pharmacological suppression of TGFβ signaling with small molecule inhibitors suppressed the acquisition of CSC properties and prevented therapeutic resistance and tumor relapse in TNBC PDX mouse models [94]. Finally, in glioblastoma (GBM), there is often an induction of aberrant TME signaling following conventional cancer therapy such as ionizing radiation and chemotherapy. For example, the pro-inflammatory cytokines IL-6 and OSM, along with their major transcription factor Signal Transducer of Activated Transcription 3 (STAT3), drive the acquisition of CSC properties resulting in therapeutic resistance. Despite the ability to target and successfully kill rapidly proliferating non-CSCs, these aggressive brain tumors relapse and effectively resist DNA damage-induced apoptosis. Interestingly, suppression of IL-6 or OSM induced STAT3 signaling in GBM following conventional therapy suppressed tumor growth and stemness-related factors, respectively [95,96]. Similarly, inhibiting STAT3-mediated signaling with a small molecule inhibitor (BBI608) resulted in suppressed stemness-related genes including Nanog, Sox2 and OCT4 which are often upregulated following conventional cancer therapy. Importantly, inhibition of these STAT3 targets suppressed tumor growth and metastasis in a variety of cancers including pancreatic, head and neck and colorectal [97]. Taken together, these data suggest that TME factors, induced by chemotherapy, may drive the TME to promote CSC plasticity and therapeutic resistance (Figure 2).

## 6. Conclusion: Targeting CSC Plasticity to Suppress Therapeutic Resistance

Following chemotherapy, the presence of residual disease at the time of surgery is associated with poor survival and early recurrence [3,4,5,96]. Unfortunately, even second-line therapies do not significantly improve outcomes for these patients, and limited data exists regarding which pathways should be targeted in patients with residual disease. There is significant support for the role of CSCs in therapeutic resistance and tumor recurrence [7,20,40]. However, while invasive CSCs are established drivers of metastasis and recurrence, our understanding of the molecular forces that regulate the generation and sustainability of CSCs remains limited. Novel therapeutic strategies have been proposed which combine conventional cancer therapies with specific inhibitors targeting CSCs [97,98,99,100,101,102,103,104,105,106]. However, this strategy may only be effective at killing pre-existing CSCs, failing to prevent non-CSCs from acquiring CSC-like properties in response to the TME and genotoxic therapy. The increasing recognition of the importance of E-M and CSC plasticity in therapeutic resistance and tumor recurrence makes the pathways governing plasticity highly attractive. Of course, targeting plasticity presents new challenges, such as the identity of which pathways should be targeted in individual tumors and the timing of the plasticity-targeting therapy. As described above, several important signaling cascades activated by TME cytokines have been identified that can promote CSC plasticity and tumor survival following chemotherapy (Figure 2). Each of these may be targetable as an adjuvant therapy to help suppress the emergence of CSC properties during conventional cancer therapy. Cytokine receptors are engaged by extracellular ligands, and, therefore, prevention of the cytokine/receptor interaction using antibodies or soluble decoy receptors is both rational and justified. Studies to determine which cytokines support CSC generation and their maintenance and how they can be targeted will provide important information about preventing CSC plasticity, which can be translated into clinical interventions to suppress metastatic progression and recurrence.
